# Synthesis of a Room-Temperature Curable Acrylic-Urethane Polymer Binder for Road Markings with High Transmittance

**DOI:** 10.3390/ma16031322

**Published:** 2023-02-03

**Authors:** Won-Bin Lim, Ju-Won Kim, Ju-Hong Lee, Ji-Hong Bae, Jin-Gyu Min, PilHo Huh

**Affiliations:** Department of Polymer Science and Engineering, Pusan National University, Busan 46241, Republic of Korea

**Keywords:** acrylic-urethane binder, room temperature curing, redox initiator system, road marking paint

## Abstract

Triol acrylic-urethane (t-AU) was synthesized from an addition reaction using trimethylolpropane, hexamethylene diisocyanate, and 2-hydroxyethyl methacrylate. The novel acrylic-urethane polymer was applied to a high-performance binder to prepare a reliable road marking paint. Acrylic-urethane polymer binder formulations were designed to optimize the effect of t-AU on the physical properties. The t-AU content in the formulation affected the adhesion and optical properties. The improvement in the adhesive performance and transparency ability for road markings was attributed to the optimal chemical structure or design of the acrylic-urethane polymer. The synthesis of t-AU was confirmed by Fourier transform infrared spectroscopy, and molecular weight and polydispersity index (PDI; PDI = M_w_/M_n_) measurements. The tensile and shear strength, hardness, gel fraction, crosslink density, contact angle, and transmittance of the acrylic-urethane polymer binder (AUP) were evaluated by curing at room temperature using a redox initiator system. An optimized AUP by adding 5 wt.% t-AU provides a viable alternative to high-performance binders in road marking paints.

## 1. Introduction

Road markings are one of the essential safety features of modern roadways with high traffic [1,2,3,4,5]. In addition to the environmental safety of the road markings, ease of application, good durability, and high functionality should be primarily considered [6,7,8,9,10]. In various road environments, road marking paints based on acrylic resin have limitations that make its drying more difficult on busy roads and can lead to traffic accidents [11,12,13,14]. Traffic marking paint-based acrylic resin could also be used with the remaining portion of the paint layer in an undried state. Some topics on the effects of the external environment and drying process on road marking paints have emerged only recently. Hence, research in recent years has focused on preparing acrylic resins with fast drying times and improved properties in the summer–winter season [15,16,17]. Duan et al. used a novel acrylic resin as a binder of waterborne printing ink with good adhesion and water resistance [18].

Acrylic-urethane binders could be employed to provide high-performance marking paints [12,19,20,21,22]. They must offer high transparency, hydrophobicity, toughness, and adhesion for used in acryl resin-based marking paint with outstanding durability and protective properties [23,24]. The basic formulations used in acrylic-urethane are acrylic monomers, soft blocks of polyols, and hard blocks of diisocyanates [25,26,27]. The adhesive properties of acrylic-urethane binders can be controlled by its hard segments and acrylic groups. Acrylic functions with carboxyl groups can be used as cross-linking sites. Therefore, the cohesion and adhesion properties of acrylic-urethane binders can be controlled by the acrylic-urethane content. Durable properties of acrylic-urethane binders can be provided by a combination of soft segments and additives, such as plasticizers and fillers. As a result, the final properties of an acrylic-urethane polymer binder (AUP) might be controlled by combining optimal amounts of these components.

As the properties and performance of polymers were influenced by their molecular weight (MW), AUP should be utilized with the optimized MW and narrow molecular weight distribution (MWD) [18,28,29,30,31]. This study examined the optimal molecular structure and produced novel AUPs with an effective curing system. Trimethylolpropane ethoxylate was selected to increase the transmittance of the acrylic-urethane binder as a trivalent polyol [32,33,34]. Hexamethylene diisocyanate without a benzene ring, as an isocyanate, was used to reduce the yellow factor. 2-hydroxyethyl methacrylate (2-HEMA) was added to impart an acrylate function to the triol urethane, and poly(methyl methacrylate) (PMMA) was used as the main acrylate of the acrylic-urethane binder to improve the physical properties. Another goal of this study was to develop a redox initiator system (ROIS) using N,N-bis(2-hydroxyethyl)-paratoluidine (PTE) with amine/benzoyl peroxide (BPO), which can initiate the free radical polymerization of acrylate at room temperature [35,36,37,38,39]. ROIS with BPO and PTE was used to induce the free radical polymerization of PMMA with the main urethane chain of the added t-AU.

## 2. Experimental Setup

### 2.1. Materials

Trimethylolpropane ethoxylate (TMPE, Mn = 1014 g/mol, Merck KGaA), hexamethylene diisocyanate (HDI, Merck KGaA), and 2-HEMA (TCI) were purchased and used after 12 h vacuum drying for dehydration. PMMA (Mn = 28,000), benzoyl peroxide (BPO), and nitrogen catalyst (PTE) were ordered from Jeongseok Chemical Co., Ltd. and vacuum-dried prior to use.

### 2.2. Synthesis of Triol Acrylic-Urethane

**Scheme 1:**
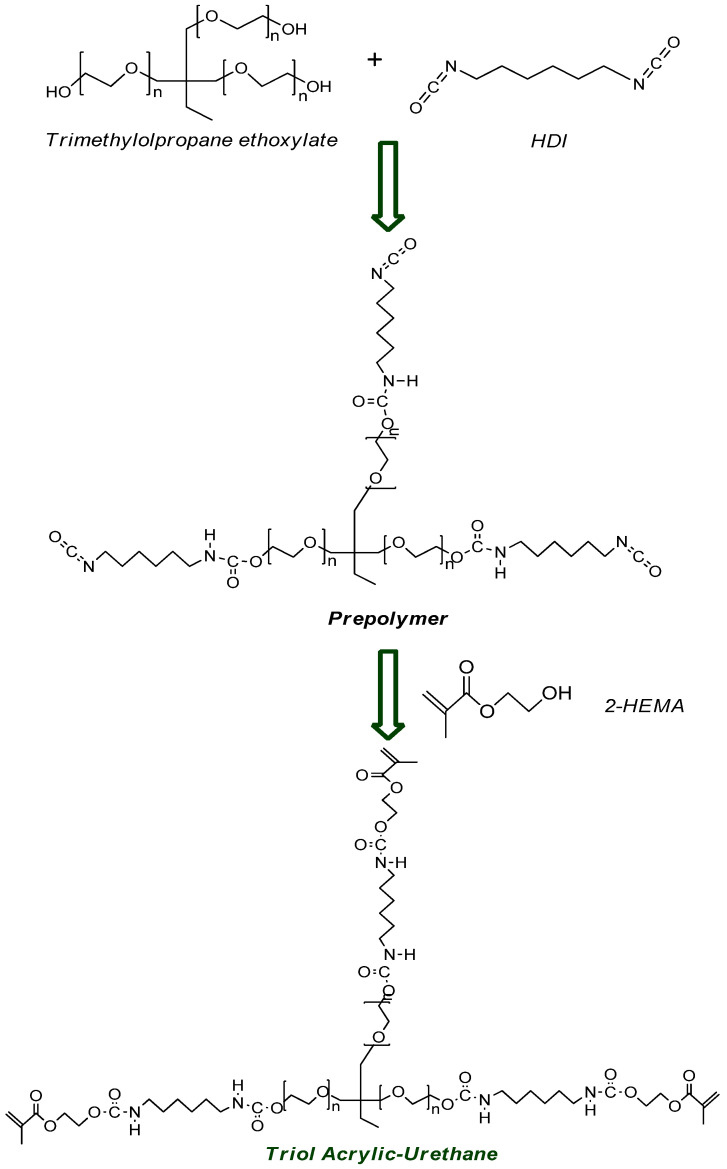
Synthetic procedure to prepare a triol acrylic-urethane resin.

Scheme 1 presents a schematic of the procedure used to synthesize a triol acrylic-urethane (t-AU).

A t-AU series was synthesized according to the following process. TMPE and HDI were charged into a 250 mL four-necked round bottom flask equipped with a thermometer, condenser, mechanical stirrer, and nitrogen purging system. The mixture was heated to 50 °C with a small amount of tin catalyst to form a prepolymer. After 3 h reaction, 2-HEMA was added as a co-monomer, and the mixture was stirred vigorously to form a 2-HEMA-terminated t-AU.

### 2.3. Room-Temperature Curing of Triol Acrylic-Urethane Polymer Binder

**Scheme 2:**
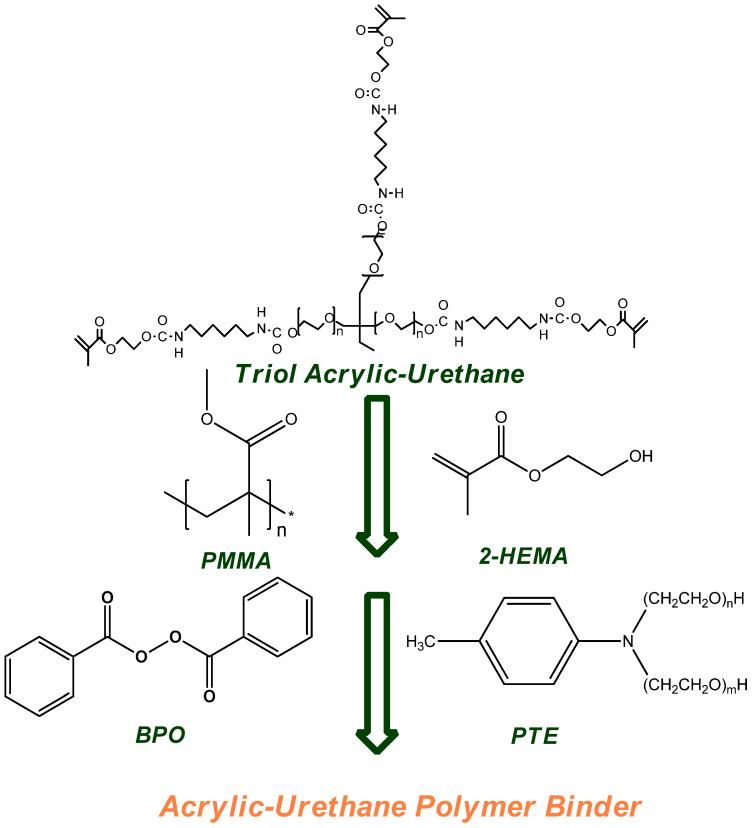
RTC of an acrylic-urethane polymer binder by mixing triol acrylic-urethane and additives.

Scheme 2 presents a schematic procedure for room-temperature curing (RTC) of acrylic-urethane polymer binder. The acrylic-urethane polymer binder (AUP) was prepared by blending triol acrylic-urethane (t-AU, content: 0–20 wt.%), PMMA, and 2-HEMA and, then, by adding PTE and BPO. The mixed polymer binder was inserted into a glass beaker; then, 1 wt.% BPO and 1 wt.% PTE were added, and mixture was mixed well. The well-mixed t-AU series was poured into polytetrafluoroethylene molds to obtain the polymer samples. Table 1 lists the composition ratio of the polymer binder.

### 2.4. Characterization

Fourier-transform infrared (FT-IR, Spectrum two, Perkin Elmer, Waltham, MA, USA) spectroscopy of the synthesized t-AU was performed with a resolution of 4 cm^−1^ over a spectral range of 400 to 4000 cm^−1^. The number average molecular weight (Mn), weight average molecular weight (Mw), and polydispersity index (PDI) of the synthesized t-AU were measured by gel permeation chromatography (GPC, Waters 2414, Waters, Milford, CT, USA). The tensile strength was measured at a rate of 10 mm/min by manufacturing a specimen according to the ASTM D638 method. The shear strength was measured at a rate of 1.3 mm/min by preparing a specimen according to the standard of the ASTM D1002 method [40]. The tensile strength and shear strength were investigated using a universal testing machine (UTM, LRX plus, LLOYD INSTRUMENT, Bognor Regis, UK). The hardness of the prepared polymer binder was measured by manufacturing a specimen according to the ASTM D2240 standard, and a durometer (A-ASKER, Kobunshi Keiki Co., Ltd., Kyoto, Japan) was used. The prepared mechanical property samples were used 24 h after manufacture, and each physical property was measured five times, with the average used for further analysis. A 5 g sample of the binder was filmed, and the gel rate of the binder was checked using the gravimetric method. The 5 g of binder film was placed in a 50 mL vial, and 30 g of tetrahydrofuran (THF, Merck KGaA) was added as a solvent. The resulting mixture was shaken for one minute, left to stand for 24 h, and shaken again for one minute [27]. After repeating this process three times, it was filtered with a vacuum pump and dried at room temperature for two hours [41]. Subsequently, the gel rate was measured by changing the weight of the prepared sample. The samples for the transmittance and contact angle measurements were prepared using a 175 μm thick applicator on a glass substrate. The transmittance of the binder was measured in a wavelength range from 300 nm to 700 nm at room temperature using an ultraviolet (UV)-vis spectrophotometer (Mega-800, Scinco, Seoul, Republic of Korea) [42]. The contact angle with water was measured at room temperature with a contact angle meter (Pheonix300, SEO, Suwon, Republic of Korea) [43].

## 3. Results and Discussion

**Figure 1:**
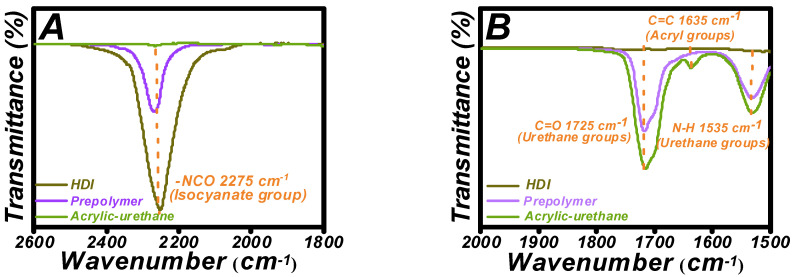
FT−IR spectra of triol acrylic-urethane by a reaction of NH groups (**A**) and C=O groups (**B**) and by the addition of 2-HEMA.

The synthesis results of triol acrylic-urethane (t-AU) were confirmed by FT-IR spectroscopy. Figure 1 presents the chemical structures of t-AU before and after acryl termination. The absorption peak of the –NCO groups at 2275 cm^−1^ disappeared gradually under the reaction process in Figure 1A, while the binding peaks at 1725 cm^−1^ and 1535 cm^−1^ due to the interaction between the –NH groups and the carbonyl groups simultaneously emerged, as shown in Figure 1B. The specific peak at 1635 cm^−1^ could be formed upon the addition of 2-HEMA, which may be due to the formation of acrylic double bonds in the urethane backbone. The results can be indicative of the occurrence of triol acrylic-urethane (t-AU) by two consecutive reactions.

**Figure 2:**
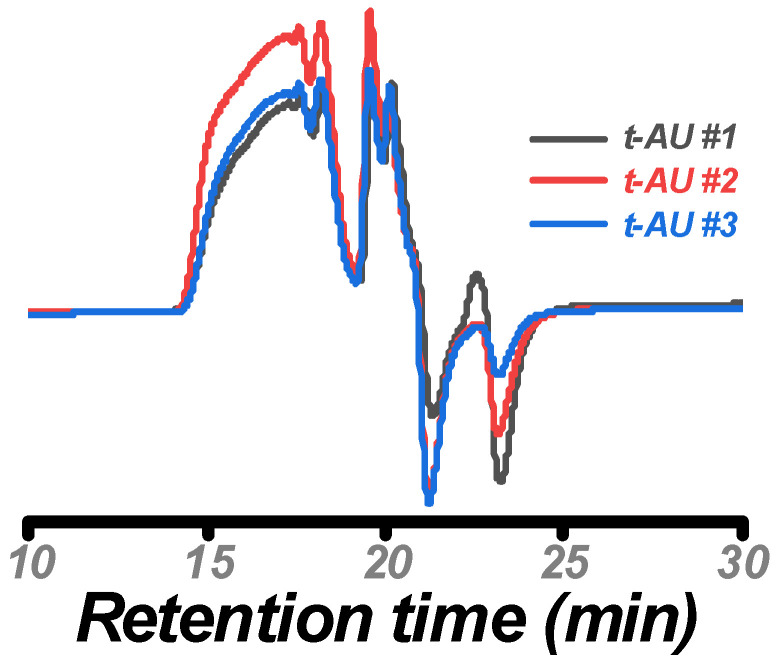
GPC curves of a triol acrylic-urethane (t-AU) series synthesized based on the same MW formulation.

The influences of the triol acrylic-urethane (t-AU) content upon the physical and mechanical properties were studied on the same molecular weight (MW). The MW can be a crucial factor affecting the properties of acrylic-urethane polymer binder (AUP). Synthesizing t-AU of a uniform molecular weight and a constant polydispersity was a must have before using it as an AUP. Figure 2 presents the GPC curves of the t-AUs synthesized under the same reaction condition, and the data are listed in Table 2. The average MW of the three t-AUs was approximately 10,776, and their polydispersities ranged from 1.96 to 2.15. This result suggests that the effect of MW on t-AU properties is limited within a constant value, except for the t-AU content.

**Figure 3:**
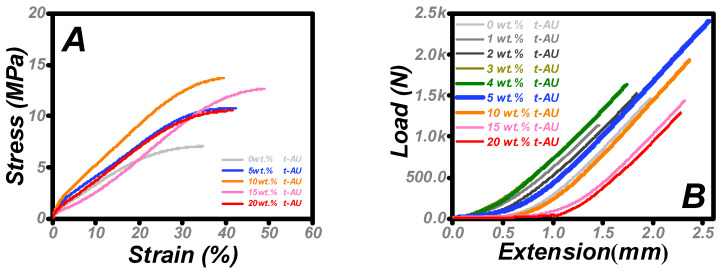
Tensile strength (**A**) and shear strength (**B**) of AUPs as a function of the t-AU content.

The effects of the chemical structures on the physical and mechanical properties of a t-AU series were studied in more detail at the same MW, based on a function of t-AU content. Figure 3A shows the measured tensile strength of the AUP series according to the difference in t-AU content. When 10 wt.% was added, the highest tensile strength appeared, and when t-AU in amounts greater than 10 wt.% was added, the tensile strength was reduced, thus being considered a defect. Figure 3B shows the measured shear stress of AUPs with different t-AU contents. As the t-AU content increased, the shear strength increased to a limiting value of 5 wt.% t-AU and, then, decreased gradually. The observation may be due to the strong hydrogen bonding and low steric hindrance by the formation of an optimized chemical structure. This result may also be because 5 wt.% t-AU can provide significant reactive groups at the molecular chain of AUP to allow for an enhanced three-dimensional network.

**Figure 4:**
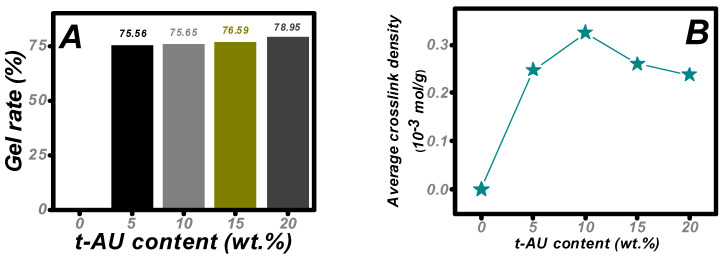
Gel rate (**A**) and average crosslink density (**B**) of AUP series based on a function of t-AU content.

The gel fraction and average crosslink density were measured to confirm the curing degree of the AUP at room temperature. The average crosslink density of the AUP series was obtained according to Equations (1) and (2).

(1) $$\sigma =\frac{1}{A}{\left(\frac{\partial ∆F_{el}}{\partial l}\right)}_{T,V}=P_{p}\gamma_{e}RT\left(\alpha -\alpha^{-2}\right)$$

where σ and A represent the tensile stress and the area of the specimen, respectively; α represents the elongation at break; and Pp is the density of the cured sample. The Helmholtz free energy (ΔF_el_) of the crosslinked network can be calculated as follows.

(2) $$∆F_{el}=\frac{1}{2}\gamma_{e}RT\left(\lambda_{x}^{2}+\lambda_{y}^{2}+\lambda_{z}^{2}-3\right)$$

where γ_e_ is the crosslink density, λ_i_ (i = x, y, and z) represents the elongation in three dimensions, T is the ambient temperature, and R is the universal gas constant [24,44]. Figure 4 shows the gel fraction and average crosslink density of AUP with different t-AU contents. The gel fraction of the AUP series crosslinked with the addition of t-AU was approximately 75–79%, as shown in Figure 4A, showing a tendency to increase as t-AU was added in excess, without a significant difference. As shown in Figure 4B, the average crosslinking density tended to increase with the addition of t-AU and reached the highest value. The average crosslinking density was calculated according to the above formula, and the data used are listed in Table 3. The binders were prepared by increasing the crosslink densities, which can be attributed to the improved transmittance and mechanical properties.

**Figure 5:**
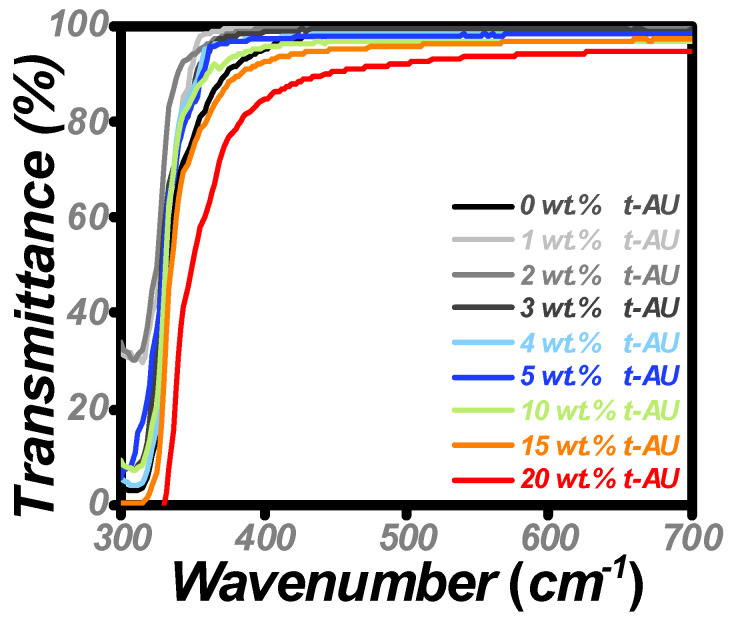
Transmittance of the AUP series as a function of t-AU content.

The AUP for road marking paint should have good optical clarity to ensure the effective absorbance and high reflection of road paint bead. Figure 5 shows the UV-visible spectra of the AUP series with different t-AU contents. The transmittance values of most AUPs were more than 90%, showing excellent transmittance in the range from 360 to 700 nm. This transmittance can guarantee that the light effectively reaches the bead. As shown in Figure 5, the increase in t-AU content leads to a decrease in transmittance. The result suggests that the t-AU amount ranging from 10 wt.% to 20 wt.% may exceed the marginal distribution of t-AU content into the APU chemical microstructure.

**Figure 6:**
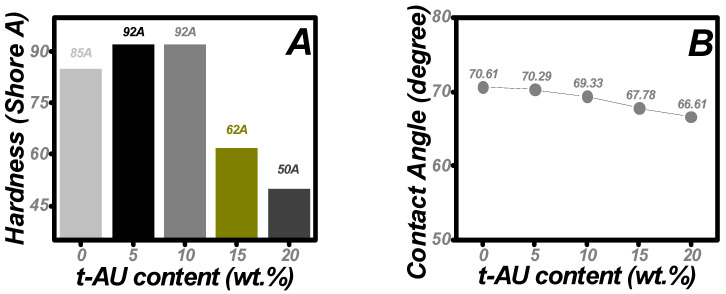
Hardness (**A**) and contact angle (**B**) of AUP series as a function of t-AU content.

In addition to the ease of application of traffic paints, durability and functionality in external environments are primarily considered. The road marking paints must maintain consistent application properties and provide a significant ‘no track’ time based on external conditions, such as higher/lower temperatures and high humidity. The dependence of the t-AU content on the durability and functionality was evaluated using the Shore A hardness and contact angle. As shown in Figure 6A, the hardness of AUP improved with increasing amounts of t-AU, up to 10 wt.%. The hardness depends on the concentration of t-AU content used. The results suggest that crosslinking in the range from 5 wt.% to 10 wt.% may be efficient because of the optimized reactive sites. Moisture adsorption depends on surface chemistry and surface-free energy. Figure 6B shows the determination of the contact angles using a contact angle meter. As the t-AU content falls below 1.0, the contact angle decreases very slowly. The t-AU content for the optimal moisture barrier appears to be 5–10 wt.%. Hence, the AUP moisture barrier may be dependent on the t-AU content in the formulation.

## 4. Conclusions

A t-AU series with the same MW was synthesized for application as a polymer binder for road marking paint. The novel formulations were prepared by tuning the t-AU content. The curing degree of AUP at room temperature was confirmed by measuring the gel fraction and average crosslink density. The adhesion strength and optical clarity of AUP were enhanced up to a limiting t-AU content and, then, decreased gradually. The tensile strength and shore A hardness also increased as t-AU was added. The improved mechanical and physical properties were attributed to the effects of an optimizing chemical structure formed in a three-dimensional chain network. Furthermore, AUP exhibited a moisture resistance property to achieve significant durability. AUP with a 5 wt.% t-AU content was applied as an excellent candidate for polymer binders in traffic road marking paint.

## Figures and Tables

**Table 1:** Split composition of the comparative groups.

Functionality	Component	Content (wt. %)				
		(a)	(b)	(c)	(d)	(e)
**acrylic compound**	PMMA	89	84	79	74	69
	2-HEMA	10	10	10	10	10
	t-AU		5	10	15	20
**catalyst**	PTE	1	1	1	1	1
**curing agent**	BPO	1	1	1	1	1

**Table 2:** Molecular weights and polydispersity index (PDI) of a t-AU series.

	*M_n_*	*M_w_*	*PDI*
**t-AU #1**	10,467	22,512	2.15
**t-AU #2**	11,040	23,491	2.13
**t-AU #3**	10,822	21,234	1.96

**Table 3:** Mechanical properties used to calculate the average crosslink density.

	Tensile Strength (MPa)	Elongation at Break (%)	Density (g/cm^3^)	Average Crosslink Density (10^−3^ mol kg^−1^)
**0 wt.%**	7.093	34.75	-	-
**5 wt.%**	10.786	42.32	0.413	0.248
**10 wt.%**	13.705	39.41	0.433	0.325
**15 wt.%**	12.681	48.71	0.404	0.260
**20 wt.%**	10.566	41.41	0.423	0.238

## Data Availability

The data presented in this study are available on request from the corresponding author.
